# Matrix‐Guided Composite Resin for Direct Diastema Closure: A Clinical Technique Report

**DOI:** 10.1155/crid/8359552

**Published:** 2026-04-17

**Authors:** Ahmad Toumaj, Vahid Cheramin, Malihe Rezaei, Mahtab Mottaghi

**Affiliations:** ^1^ Faculty of Dentistry, Department of Prosthodontics, Islamic Azad University Tehran Medical Sciences, Tehran, Iran, iautmu.ac.ir; ^2^ School of Dentistry, Islamic Azad University Tehran Medical Sciences, Tehran, Iran, iautmu.ac.ir; ^3^ School of Dentistry, Hamadan University of Medical Sciences, Hamadan, Iran, umsha.ac.ir; ^4^ School of Dentistry, Mashhad University of Medical Sciences, Mashhad, Iran, mums.ac.ir

**Keywords:** composite resins, diastema, matrix bands

## Abstract

The demand for esthetic dental procedures, particularly among younger patients, has increased significantly in recent years. Diastema, the gap between anterior teeth, is a prevalent esthetic issue. This case report introduces a new and less invasive procedure for diastema closure termed the putty matrix diastema closure (PMDC) method. A 49‐year‐old female patient presented with a diastema between the upper right central and lateral incisors. The approach utilized a custom silicone putty index and an ultrathin metal matrix band to facilitate progressive composite repair. The treatment effectively achieved superior esthetic and functional outcomes while conserving natural tooth structure and avoiding more invasive interventions like orthodontics or porcelain veneers. This case report has been prepared in accordance with the CARE guidelines.

**Summary**

• We demonstrated a new method for diastema closure introduced as the putty matrix diastema closure (PMDC) technique, which involved a silicone index and a metal matrix band.
• This technique provides a less invasive alternative for more aggressive procedures, such as porcelain veneers and orthodontics.
• This approach successfully strikes a balance between esthetic outcome, patient satisfaction, and durability.

## 1. Introduction

Nowadays, esthetics has become a top priority for the younger generations, with an increasing demand for enhanced facial and dental esthetics [1, 2]. Advancements in communication technology have significantly influenced patient’s awareness and expectations regarding the esthetic options offered in dentistry [3]. Therefore, dental specialists have expanded their skills and qualifications to meet these growing demands. Esthetic discrepancies in the anterior zone commonly present as diastema, mesiodens, fractures, microdontia, and Talon’s cusp. Among these concerns, managing diastema—the gap between two or more adjacent teeth—has become increasingly popular in the past few years [2]. Diastema is caused by factors such as frenum morphology, supernumerary teeth, and nasal airflow condensation [4]. The treatment plan for diastema closure depends on several factors, including etiology, economics, patient preferences, and time availability [5].

Several treatment plans are involved in managing diastema closure, including orthodontic treatments, crowns, laminate veneers, and direct composite restorations [6]. Orthodontic diastema closure requires applying fixed braces, which takes the most time and cost [7]. Porcelain or veneer crowns also lead to successful outcomes. However, it requires multiple sessions and is significantly more expensive than composite resin. Furthermore, the preparation of the intact enamel is often essential in ceramic restorations [5]. On the other hand, direct composites in operative dentistry could create a highly natural diastema restoration in a single appointment [8]. The primary benefits of direct procedure are cost‐effectiveness, simplicity, predictability, and efficiency [7]. However, the direct approach requires considerable finishing and polishing skills to prepare a highly polished restoration with perfect anatomic form without a black triangle in the gingival quadrant [8]. This approach proposed new opportunities for minimally invasive dentistry, as tooth shape, position, and color could be improved without removing any major tooth structure [6]. Studies have shown that direct composite build‐ups applied for anterior tooth recontouring and space closure have an overall 5‐year survival rate of 84.6%, with over 90% of the restorations rated as clinically excellent or good. Furthermore, any failed restorations were effectively repaired, achieving a 100% functional survival rate [9]. Despite the many advantages of direct composite restorations, they become challenging and technique‐sensitive in cases of severe malformation. Hence, we could use additional equipment to simplify the direct application of composite materials, such as matrix bands and silicone indexes [9].

The introduction of various matrix techniques optimized the functional and esthetic results of freehand composite restorations in the anterior teeth [10]. The application matrix band has been reported to simplify the creation of the proximal anatomy, but more is needed to ensure the proper width in the anterior teeth. We could use a silicone index to guarantee the appropriate anterior teeth width [5].

In current restorative dentistry, silicone indexes are highly useful for various purposes, from treatment planning to tooth preparation and final restorative stages [11]. According to previous studies, silicone‐based index approaches could cause several complications, including a sharp emergence profile and composite overlaps [12]. These complications are due to problems in the fabrication of the silicone index. The silicone index from the wax‐up does not provide the exact replication of the cervical anatomy. However, the gingival third details of the silicone index are less significant for the restorations extending from the middle third to the incisal border; they are crucial for diastema closure [4]. As a result, the major complication is the inability to transfer the precise gingival anatomy to the wax‐up. To overcome this, we eliminated the wax‐up stage in this report.

This report demonstrated an approach for diastema closure introduced as the putty matrix diastema closure (PMDC) technique, emphasizing its advantages and clinical considerations for effective diastema management. Through meticulous tooth preparation, accurate measurements of interdental space, and strategic application of the putty wash and composite resin, this approach offers a minimally invasive and time‐saving solution that fulfills both functional and esthetic requirements.

## 2. Case Presentation

A 49‐year‐old female patient presented to the dental clinic with a chief complaint of noticeable space between her top anterior teeth. The patient exhibited systemic health, with no history of systemic diseases, medication use, or allergies. The dental history indicated an absence of prior orthodontic or restorative treatments in the affected area. The patient requested a conservative and esthetically acceptable treatment that would avoid orthodontic or indirect restorative approaches.

## 3. Clinical Findings

The intraoral examination identified a 1.8 mm diastema between teeth #11 and #12, along with cervical caries on tooth #11. The gingival tissues exhibited a healthy appearance, without signs of inflammation or periodontal disease. The patient’s occlusion was stable, and her oral hygiene appeared to be satisfactory.

## 4. Timeline

During the initial visit, the patient received consultation, diagnosis, and treatment planning. The subsequent day, the clinical procedure was conducted, encompassing tooth preparation, gingival retraction, and composite restoration via the PMDC technique. A follow‐up evaluation conducted 3 months later confirmed the stability of the restoration and the presence of healthy gingival conditions.

## 5. Diagnostic Assessment

The diagnosis of localized diastema and cervical caries was confirmed through clinical examination and intraoral photography. The radiographic assessment revealed no periapical or periodontal abnormalities.

## 6. Clinical Procedure

This specific technical modification for diastema closure was conducted by employing a putty‐based palatal matrix and an ultrathin metal band to improve contour control and preserve gingival tissue.

### 6.1. Teeth Preparation

**Figure 1:**
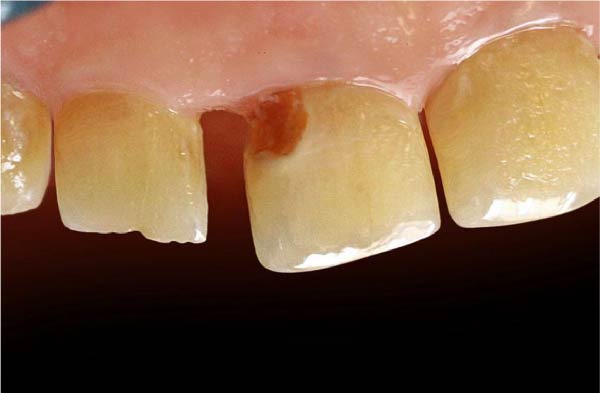
An illustration of a diastema between the upper right central and lateral incisors and a cervical decay in the central incisor.

**Figure 2:**
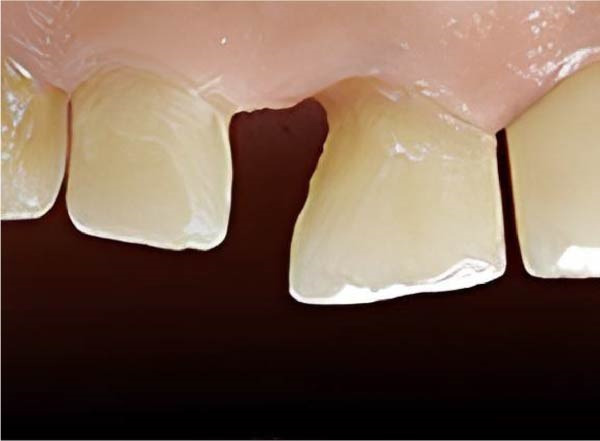
The prepared teeth post removal of old caries.

A diastema between the upper right central and lateral incisors and a cervical decay in the central incisor were detected during the clinical examination (Figure 1). All carious lesions, plaque, and calculus were thoroughly removed from the proximal surfaces of the teeth using an ultrasonic scaler (Guilin Woodpecker Medical Instrument Co. Ltd.) and high‐speed dental instrument (Figure 2). The proximal surfaces were then roughened using a fine‐grit, flame‐shaped diamond burr at low speed to promote composite bonding. Due to the diastema location, rubber dam isolation was not feasible. Instead, cotton rolls and high‐volume suction were applied to ensure moisture control throughout the whole process.

### 6.2. Evaluation of Interdental Space

**Figure 3:**
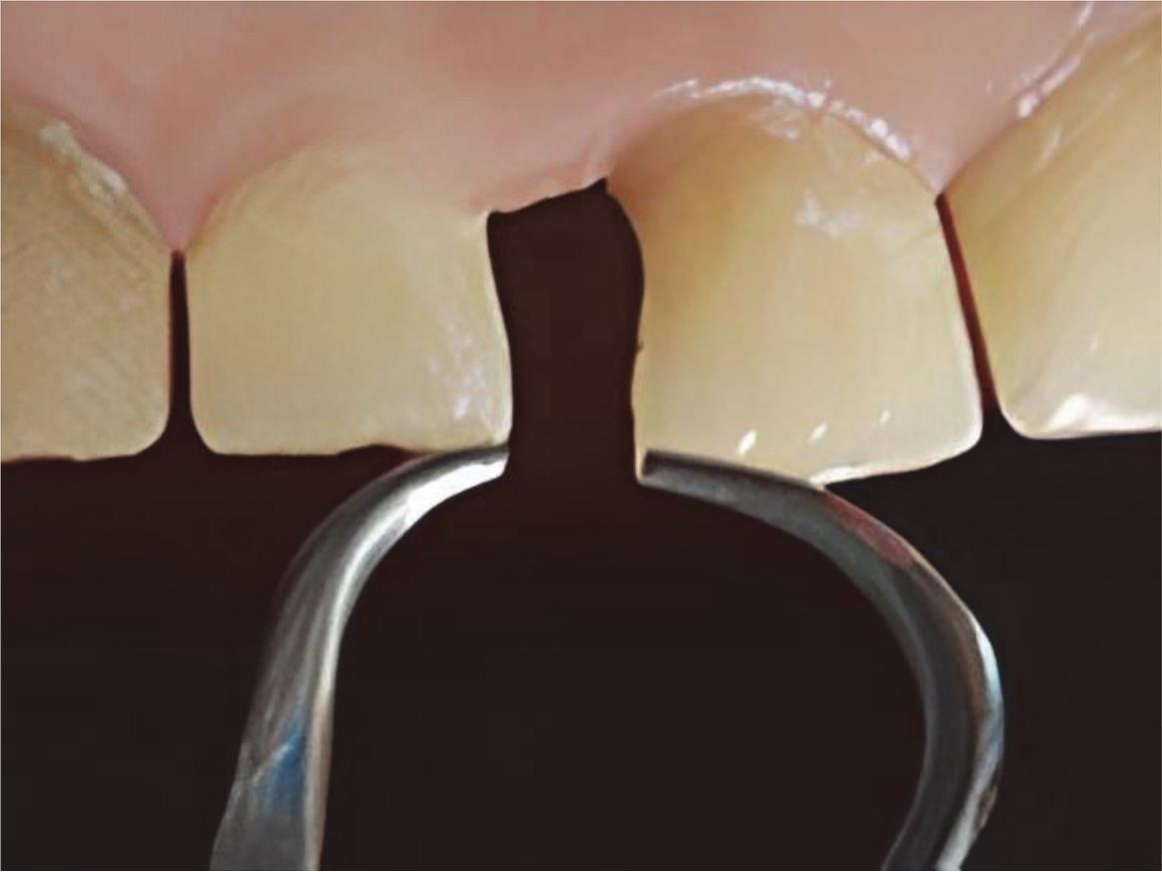
Assessment and measuring the proximal space using a dental caliper.

We precisely measured diastema space using a calibrated dental caliper (Hilbro, precise to 0.1 mm) (Figure 3).

### 6.3. Gingival Retraction and Impression Preparation

**Figure 4:**
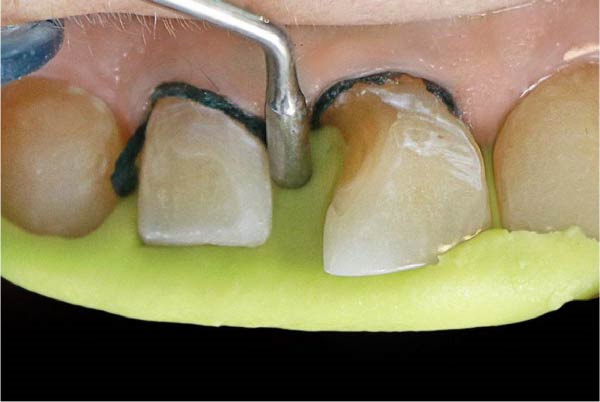
Pressing the putty material from the palatal surface to create a silicone index and packing the unset proximal putty to the proper shape.

The subgingival retraction was achieved using a #000 nonepinephrine retraction cord (Retreat K Henry Schein, Melville) placed circumferentially around the teeth. The region was rinsed with isotonic saline and an antiseptic mouthwash. Afterward, a silicone‐based putty material is carefully applied to the lingual surface, extending over the diastema. A condenser is used from the labial surface within the diastema space to shape the unset putty, replicating the natural tooth thickness and contour (Figure 4).

### 6.4. Disinfection and Matrix Delineation

**Figure 5:**
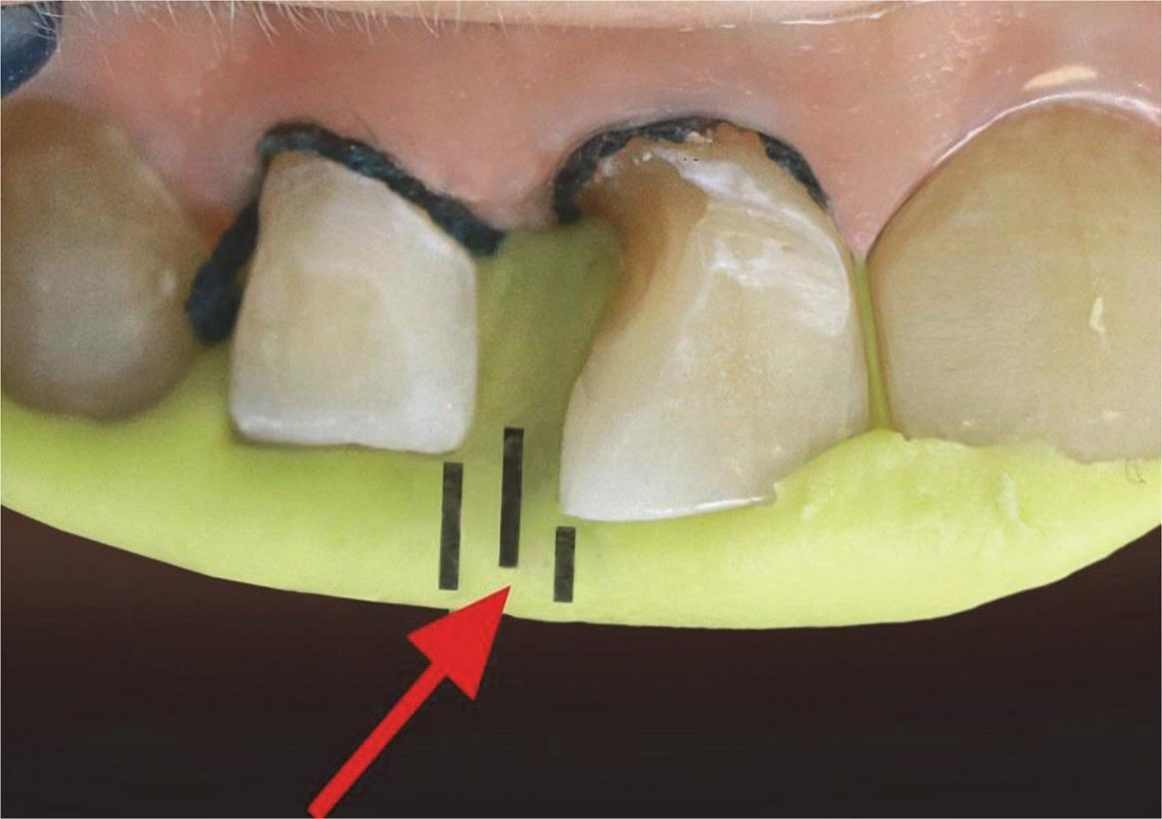
Dividing the interproximal space to define the ideal location of the proximal wall.

**Figure 6:**
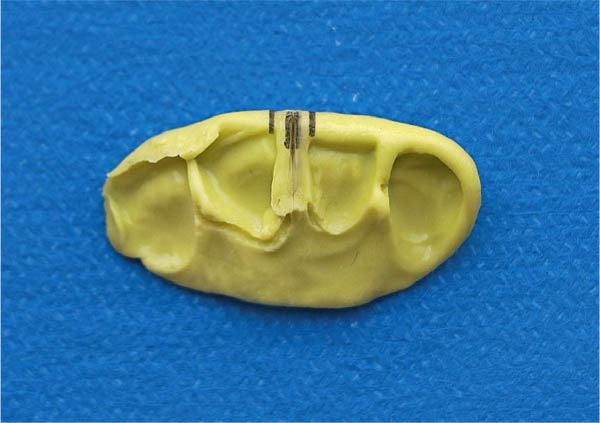
The silicone index was taken from the palatal surface of the upper right central and lateral incisors.

**Figure 7:**
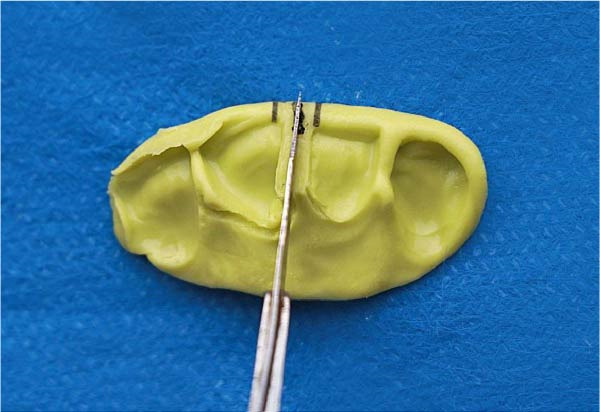
An incision was made in the proper location to create a space for an ultrathin metal matrix band.

We applied a 0.2% chlorhexidine solution to disinfect the internal surface of the diastema. Once the putty material had been set, three parallel delineation lines were inscribed on the putty surface in the interdental region, according to the teeth’s esthetic evaluations, for precise matrix placement (Figure 5). The putty matrix was carefully removed from the palatal surface in the patient’s mouth (Figure 6). The ideal placement was incised using a No. 11 surgical blade (Figure 7).

### 6.5. Matrix Band Insertion

**Figure 8:**
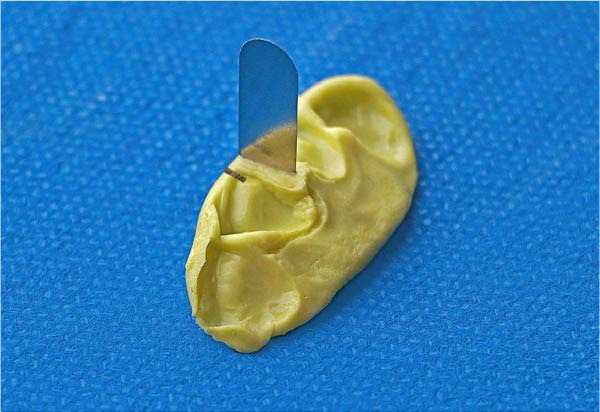
The ultrathin matrix band is placed in the proximal putty so that half of its width is embedded into the proximal surface.

**Figure 9:**
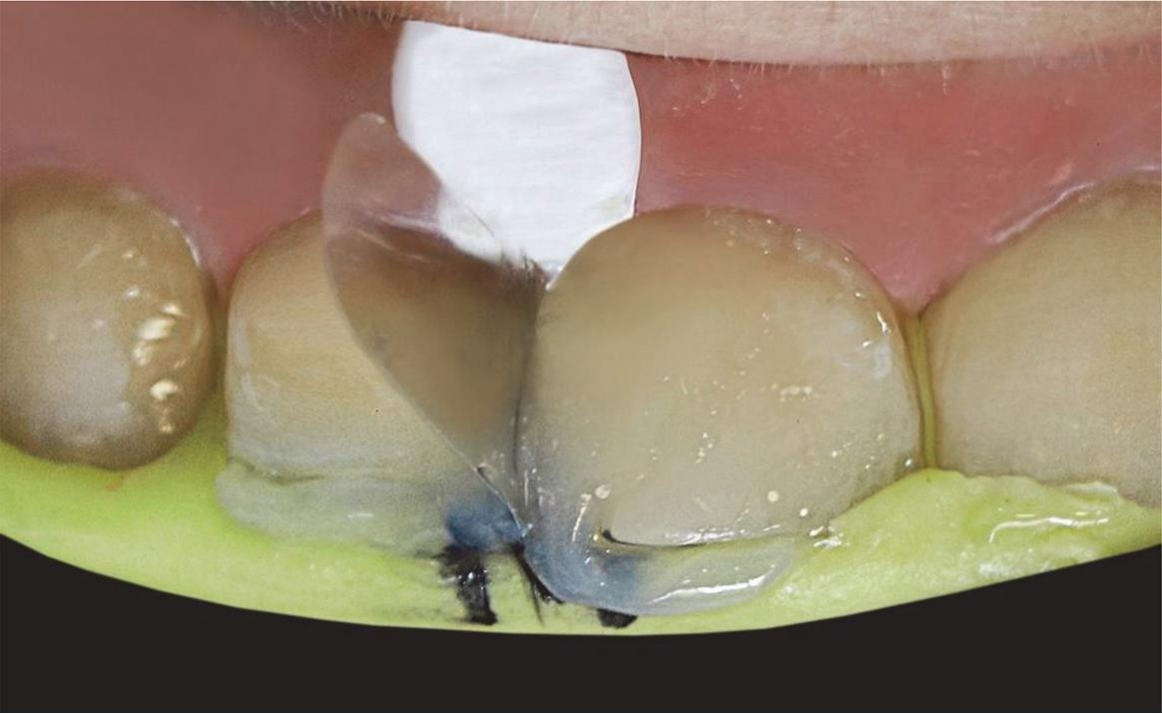
Placement of the complex of the putty and matrix in the mouth with improved fixation by curing transparent composite on both sides of the matrix.

An ultrathin metal matrix (Tofflemire, 0.0015 inches) was placed in the proper position within the putty index with minimal movement. We inserted the band vertically with 0˚ angulation, embedding 1 mm of width into the putty matrix for stabilization while maintaining anatomical contour (Figure 8). To ensure stability throughout the process, a small amount of transparent composite was applied on both sides of the matrix band, allowing it to adhere lightly to the putty surface (Figure 9).

### 6.6. Isolation and Bonding Preparation

We covered the internal surface of the putty index with glycerin gel to preserve the optimum surface hardness and color stability during composite curing by preventing the oxygen inhibition layer. Teflon tape was placed around the gingival margin to create an isolated environment and prevent gingival fluid seepage. In the subsequent stage, the enamel of maxillary central and lateral incisors is beveled, followed by selective etching of only the enamel with a 37% phosphoric acid solution for 15 s, after which it is washed and air‐dried. Next, a self‐etch adhesive is applied to the area according to the product brochure’s instructions and is light‐cured.

### 6.7. Composite Application and Final Restoration

**Figure 10:**
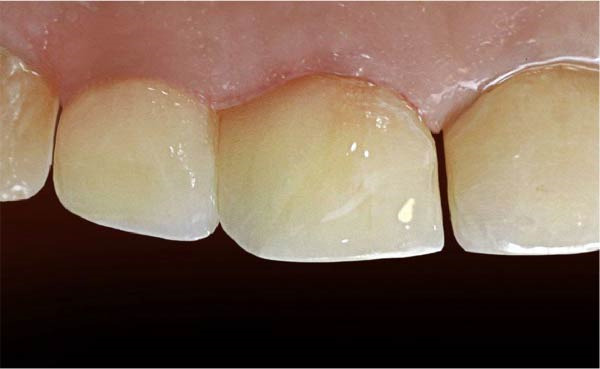
The patient’s mouth after finishing and polishing.

A nanohybrid composite resin was applied incrementally, while the putty matrix index provided palatal support in the packing process. Initially, a translucent composite shade (A2) was used to create a thin lingual shelf, which served as the foundation for the diastema closure. Subsequently, additional increments of composite were added to fill the gap. The restoration was light‐cured for 40 s per increment. Once the polymerization process was finished, the PTFE and retraction cord were removed carefully. Following completion of composite build‐up and initial polymerization, the ultrathin metal matrix band was removed while the retraction cord remained in place to maintain gingival displacement during contour refinement. After final curing and finishing procedures, both the PTFE tape and retraction cord were carefully removed. The marginal excess and incisal height were modified using a diamond burr. The polishing process was conducted using a handpiece, interproximal strip, polishing rubber, and brushes loaded with polishing paste to create a glossy appearance. The goal of this meticulous polishing process was to ensure long‐term visual appeal by preventing plaque build‐up and discoloration. Figure 10 depicts the final appearance of the restoration.

## 7. Follow‐Up

**Figure 11:**
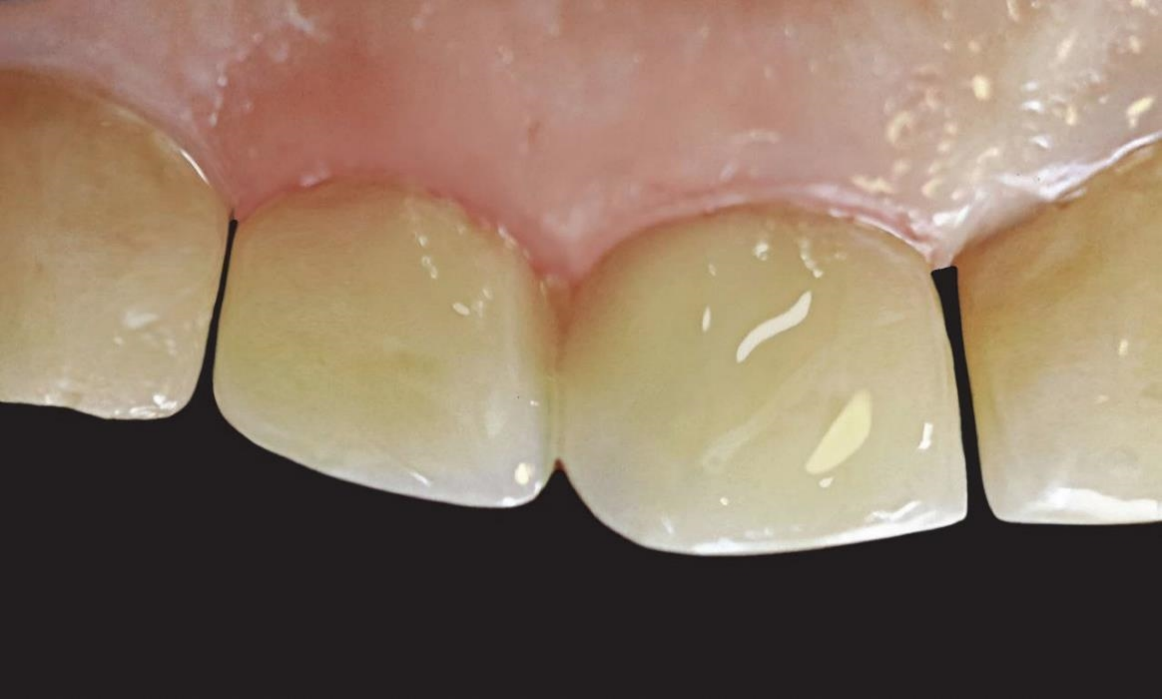
Three‐month follow‐up showing stable restoration and healthy gingiva.

A 3‐month follow‐up was performed to evaluate the clinical stability of the restoration and the condition of the gingival health. High‐resolution intraoral images were acquired (Figure 11). The gingival evaluation indicated normal color, shape, and consistency, with no evidence of inflammation, recession, or bleeding on probing. The restoration was preserved, and the patient reported satisfaction with the esthetic result.

## 8. Patient Perspective

The patient reported a high level of satisfaction with the outcome, indicating a notable enhancement in the appearance of her smile and a significant increase in her confidence. The patient reported comfort during the procedure, with no postoperative discomfort or complications noted.

## 9. Discussion

This report describes a new and minimally invasive approach for diastema closure called the PMDC technique. This method offers several advantages over conventional approaches, including direct composite restoration, porcelain veneer, and orthodontic therapy. The major benefit of this technique is that it creates precise, anatomically accurate, and esthetically pleasing restorations without significant enamel removal or multiple sessions. We used the putty matrix to manage and isolate the diastema space and ensure that the restorative material is placed exactly where necessary to create appropriate proximal contact.

Various studies have explored the application of silicone indexes in the diastema closure process. Kabbach et al. [5] introduced a technique involving two silicone indexes: one made from putty polyvinyl siloxane (PVS) and the other from a combination of putty and light PVS materials. This approach guarantees the width of the upper incisors and improves gingival health. Similarly, Revilla‐Leon et al. [13] demonstrated the effectiveness of a 3‐piece additively manufactured clear silicone index, which altered the insertion path from vertical to horizontal, accurately translating the size and shape of the virtually planned restoration and allowing light‐curing of the material through the silicone index. Compared to these methods, the PMDC technique improves matrix handling by integrating a single putty‐based palatal matrix with an ultrathin metal band, thereby offering precise palatal support and control over proximal anatomy without the need for multiple index pieces or complicated insertion paths.

Proximal wall design is essential in composite restorations. Matrix bands are typically used to develop the proper proximal walls in the composite restorations. Previous studies have explored several methods to create a proper proximal wall. For instance, Nodgouda et al. [2] used a sectional matrix to create the proximal walls. Kabbach et al. [5] used a stainless‐steel matrix to assist in the formation of proximal anatomy. Korkut et al. [14] used PTFE tape to create proximal contact. In the current report, proximal contact was facilitated by using both PTFE tape and an ultrathin metal matrix band. During composite placement, the metal matrix band provided additional support and facilitated identification of the marginal areas. The band’s mobility was reduced by placing it vertically and gently embedding it into the putty matrix, allowing more predictable guidance of the initial proximal contour. This approach does not claim to produce a superior final anatomy; rather, it enhances clinician control during the early stages of contour formation. It should be noted that the immediate postoperative and follow‐up photos were taken at slightly different angles, which may have altered the perceived morphology of the proximal contour and contributed to the differences between the two figures.

The subgingival retraction was conducted using a retraction cord, ensuring a dry and clean area to maximize the bonding strength between the restorative material and tooth structure. Proper moisture control is essential to the success of composite restorations, especially in cases requiring gingival margin management. While rubber dam isolation is usually applied to ensure optimum moisture control and effective gingival retraction, we could not use it in this report because the clamp would interfere with the placement of the putty index [7]. As a result, alternative isolation techniques, using cotton rolls and careful retraction by PTFE tape and cord, were employed to keep the operative field dry. While effective, these methods are fundamentally less reliable than rubber dam isolation.

Several studies have highlighted direct composite applications for diastema closure [2, 14, 15]. Nadgouda et al. [2] described two methods: Mylar strips for the palatal shelf, sectional matrix bands for the proximal walls, and prefabricated strip crowns. In another study, Soetojo et al. [15] treated white spots and central diastema with partial direct veneer restoration, which led to favorable esthetic outcomes. Korkut et al. [14] used direct composite restoration without prior preparation and in a single session. The PMDC approach builds upon these principles by providing superior control over restorative contouring, proximal contact establishment, and periodontal health. Our technique improves the predictability of functional and cosmetic outcomes by integrating an ultrathin metal matrix with a putty‐based palatal index.

The main limitation of the PMDC approach is compromised isolation. Inserting a rubber dam, which is standard for optimal moisture control and bonding, was impractical in this instance due to the clamp obstructing the putty index. While alternate isolation techniques (cotton rolls, high‐volume suction, and careful retraction using PTFE tape and cord) were used to ensure a dry field, these procedures are fundamentally less dependable than a rubber dam. Future modifications of this technology may investigate altered clamps, custom rubber dam frameworks, or sectional isolation systems to enhance moisture management while maintaining the exact positioning of the putty matrix.

The PMDC technique is indicated in patients with multiple diastemas, especially when indirect restorations are not feasible. It is also suggested that employing a rubber dam is impossible. The principal contraindications of this technique are cases with narrow diastema (≤0.5 mm) or teeth with poor prognosis. More clinical studies with long‐term follow‐ups are recommended to assess the effectiveness of the PMDC technique.

In conclusion, the PMDC technique has shown promising outcomes for diastema closure management. This new technique, which combines an ultrathin metal matrix band with a putty matrix, ensures accurate anatomical structure and optimum esthetics. It offers a minimally invasive approach that provides functional, durable, and esthetically pleasing outcomes. These results inspire further exploration of the potential of the PMDC technique in restorative dentistry.

## Author Contributions

**Ahmad Toumaj:** conceptualization, methodology, investigation, visualization, writing – original draft, writing – review and editing, project administration. **Vahid Cheramin:** investigation. **Malihe Rezaei:** investigation. **Mahtab Mottaghi:** software, writing – review and editing.

## Funding

The authors state that no funds, grants, or additional support were received in the preparation of this case report.

## Ethics Statement

This case report adheres to the CARE Guidelines, ensuring clarity, transparency, and completeness in the presentation of clinical information. Ethical clearance for this case report was obtained in compliance with the institutional policies of the Faculty of Dentistry, Islamic Azad University, Tehran Medical Sciences.

## Consent

The patient allowed personal data processing, and informed consent was obtained from the participant included in the study.

## Conflicts of Interest

The authors declare no conflicts of interest.

## Data Availability

The data that support the findings of this study are available from the corresponding author upon reasonable request.
